# Segment IV approach for difficult laparoscopic cholecystectomy

**DOI:** 10.1002/ags3.12297

**Published:** 2019-11-11

**Authors:** Hiroaki Kitamura, Shuichi Fujioka, Taigo Hata, Takeyuki Misawa, Katsuhiko Yanaga

**Affiliations:** ^1^ Department of Surgery The Jikei University Kashiwa Hospital Chiba Japan; ^2^ Department of Surgery The Jikei University School of Medicine Tokyo Japan

**Keywords:** critical view of safety, gallbladder, laparoscopic cholecystectomy, segment IV of the liver, subtotal cholecystectomy

## Abstract

Although achieving the critical view of safety (CVS) is useful for avoiding vasculobiliary injury during laparoscopic cholecystectomy (LC), the CVS cannot always be achieved in cases of severe cholecystitis because of technical difficulties. Herein, we focused on segment IV of the liver and its diagonal line (D‐line) as a feasible landmark for carrying out difficult LC. The D‐line connects the right dorsal and left ventral corners of segment IV and is used as the vectoral landmark, which is where the gallbladder is first dissected to achieve CVS without misidentification. Conversion to subtotal cholecystectomy along the D‐line is also feasible when gallbladder wall scarring is severe. We named this procedure the segment IV approach for LC. Sixty‐two consecutive difficult LC (including 27 scheduled LC after percutaneous transhepatic gallbladder drainage [PTGBD] and 35 conservatively treated cases of Tokyo Guidelines [TG] grade II cholecystitis) were managed by the segment IV approach. Successful gallbladder extraction along the D‐line was achieved in 44 (71%) cases; all of these cases also achieved CVS following total cholecystectomy. The other 18 (29%) cases were converted to subtotal cholecystectomy because gallbladder extraction along the D‐line failed as a result of severe cholecystitis with inflammatory adhesion with surrounding structures. Median operative time and intraoperative blood loss were 135 (range, 54‐290) min and 10 (range, 0‐100) mL, respectively. No intra‐ or postoperative complications were observed. The segment IV approach is feasible for achieving CVS and for considering subtotal cholecystectomy in difficult LC cases where scarring of the gallbladder wall is present.

## Graphical abstract

Schematic demonstrations of the segment IV approach. Under physiological conditions, the D‐line runs to the right border of the Hiller plate. Condition where the cystic plate is thickened and shrinking is caused by gallstones. The positional relationship between the D‐line, with respect to the Hiller plate, remains unchanged.

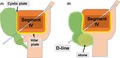

## 1 INTRODUCTION

The critical view of safety (CVS) has been proposed as a means of avoiding major vasculobiliary injury (VBI) that occurs during laparoscopic cholecystectomy (LC) and is caused by misidentification of cystic structures.^1^, ^2^, ^3^ The CVS is a technique for anatomical identification, which targets the cystic duct and the cystic artery.^4^ It has been accepted as a result of a sudden increase in the occurrence of VBI after the introduction of LC.^1^ Achievement of CVS requires dissection of the proximal one‐third of the cystic plate and skeletonization of the cystic structure; however, these tasks are not easy in the scenario of difficult gallbladder because of severe scarring around the neck of the gallbladder.^5^, ^6^ Recently, the 2018 Tokyo Guidelines (TG‐18) proposed imaging of a connecting line between the base of segment IV of the liver and the roof of Rouviére's sulcus as the appropriate first step for achieving CVS during LC.^7^ However, when managing a difficult gallbladder, a more anatomically specific landmark should be designated to achieve LC, as the “base” of segment IV provides obscure and anatomically non‐specific positional information. Rouviére's sulcus is also widely accepted as a landmark, at least in the posterior view, as it indicates the bifurcation point of hepatic inflow structures to the right hepatic lobe. However, Rouviére's sulcus is recognizable in only 75% of patients as its visibility can be obscured by omental fusion or by inflammatory changes in acute cholecystitis, precisely when it is most needed.^8^ Rouviére's sulcus is not always recognizable because of gallstones impacting the neck of the gallbladder in difficult LC. In the present study, we advocate the diagonal line of segment IV of the liver as a feasible anatomical landmark for difficult LC and as a reference for specifying gallbladder dissection.

## 2 MATERIALS AND METHODS

### 2.1 Patients

From October 2015 to December 2018, 273 patients diagnosed with cholecystolithiasis or gallbladder polyps underwent LC; among them, 192 consecutive LC including 62 difficult LC and 130 non‐difficult LC carried out by SF and KH were managed by the segment IV approach. Difficult LC was defined as cases classified as grade II cholecystitis by the TG‐18 guidelines and cases where LC was done at least 7 days after the onset of cholecystitis.^9^ Non‐difficult LC was defined the condition except the above mentioned criteria. Among the difficult LC group, percutaneous transhepatic gallbladder drains (PTGBD) were placed preoperatively in 27 cases, whereas conservative treatment without gallbladder drainage was implemented in 35 cases. All LC were electively scheduled. Patient characteristics and outcomes of the difficult and non‐difficult gallbladder procedures are summarized in Tables S1 and S2.

### 2.2 Surgical technique

All LC were carried out using the conventional four‐port method. The operator's 5‐mm working port (for the operator's right hand) was inserted at the epigastric lesion. A 5‐mm port for the operator's left hand was inserted at the right subcostal area along the right mid‐clavicular line. A 5‐ or 10‐mm flexible videoscope was inserted through the 12‐mm port that was placed at the umbilicus. For gallbladder retraction, a 5‐mm port was placed at the subcostal area along the anterior axillary line. Under pneumoperitoneum, visualization of the hepatic hilar region was provided by cranial retraction of the gallbladder fundus. After dissection of a cholecystitis‐related adhesion around the gallbladder, superficial landmarks such as Rouviére's sulcus and segment IV of the liver, the infundibulum of the gallbladder, and the common bile duct were recognized. Rouviére's sulcus is fundamentally confirmed as an essential surface landmark to ensure the D‐line lies above it (Figure S1). Alternatively, we use these findings instead of Rouviére's sulcus when its border is obscured so that the liver surface at the posterior side of the gallbladder is continuously recognized from the gallbladder fundus to the D‐line.

**Figure 1:**
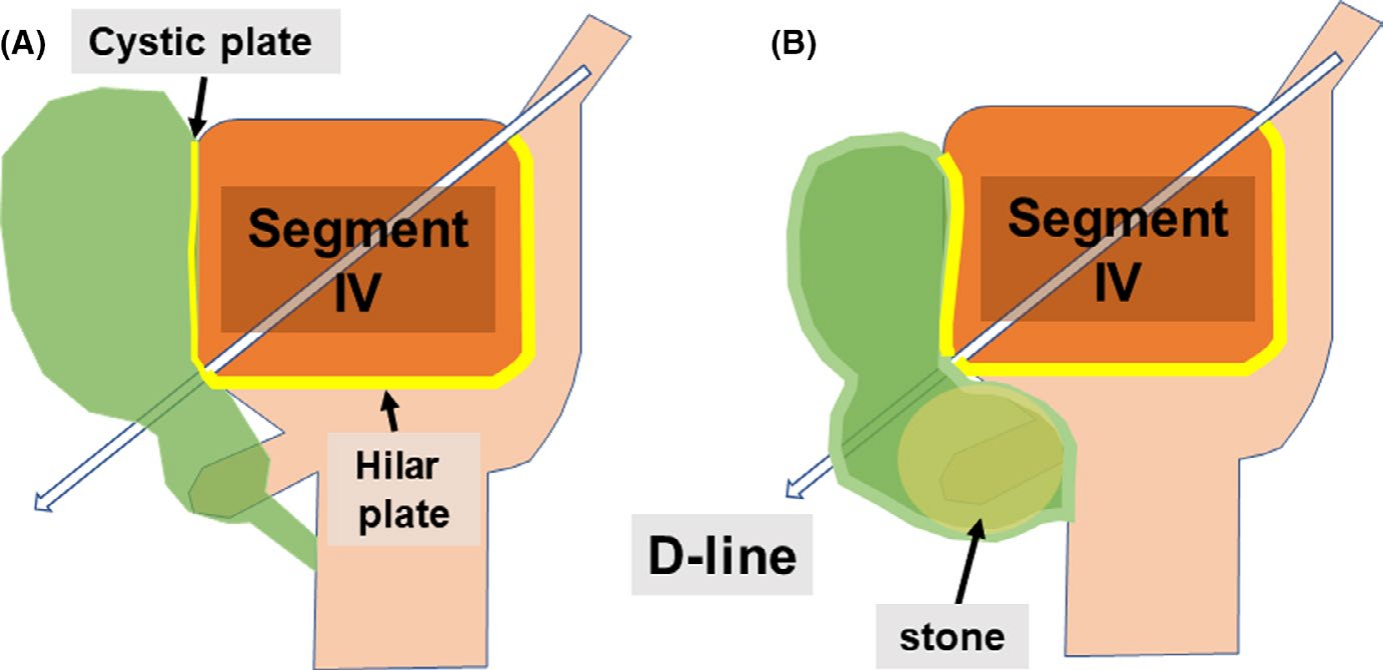
Schematic representations of the segment IV approach. (A) Under physiological conditions, the D‐line runs to the right border of the hilar plate. (B) Condition where the cystic plate is thickened and shrunk as a result of gallstones. The positional relationship of the D‐line, with respect to the hilar plate, remains unchanged. D‐line, diagonal line of segment IV of the liver

**Figure 2:**
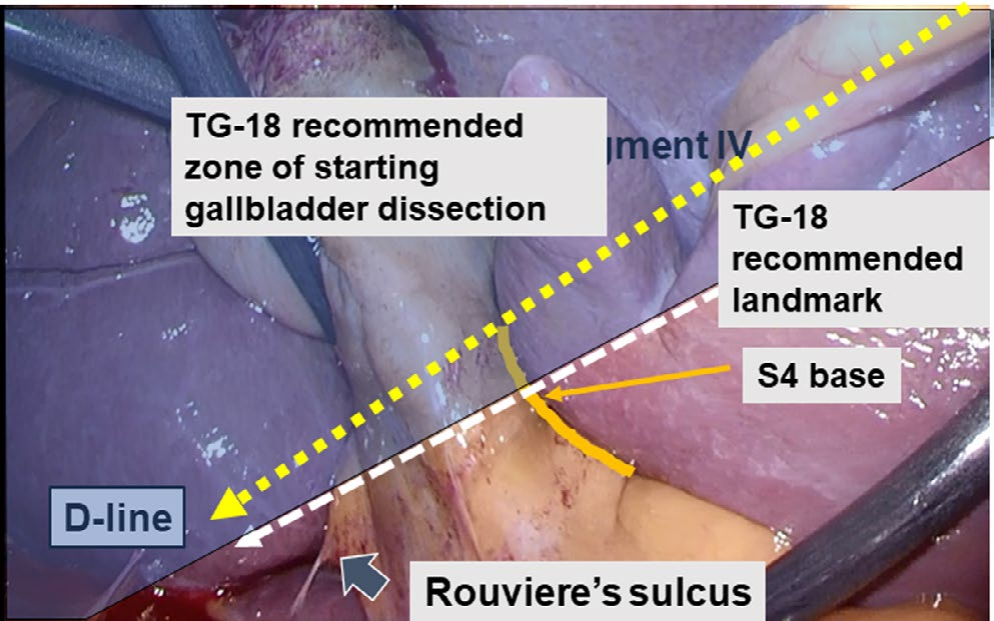
Clinical application of the segment IV approach. Diagonal line of segment IV of the liver (D‐line) is shown (yellow dotted line). The line of dissection recommended according to the 2018 Tokyo Guidelines (TG‐18) is represented as a white dotted line. D‐line, diagonal line of segment IV of the liver; S4, segment IV

The 5‐mm port for the operator's right hand was preferably placed at the highest possible position so that a working device could be inserted parallel to the caudal surface of the liver. Tying the falciform ligament, which is retracted extracorporeally through the side of the epigastric working port, enabled matching between the diagonal line of segment IV (D‐line) and the direction in which the gallbladder dissection would proceed (Figures 1 and 2).

**Figure 3:**
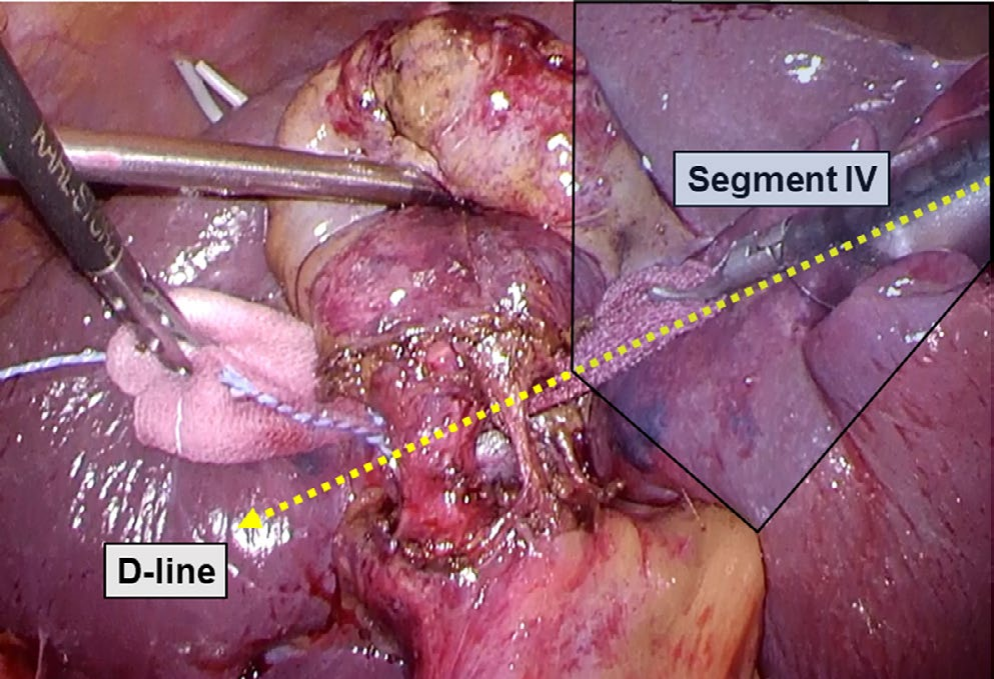
Critical view of safety (CVS) is secured using the segment IV approach. The cystic structure is dissected after isolating the gallbladder neck using surgical gauze to achieve CVS. D‐line, diagonal line of segment IV of the liver

Dissection was started by incising the gallbladder serosa at the right posterior corner of segment IV with rounded dissecting forceps along the D‐line, and dissection proceed within the subserosal layer of the gallbladder under direct vision by using a flexible laparoscope to avoid injury of the liver parenchyma. While the cystic plate (subserous layer of the gallbladder wall) meets the anterior and posterior Glissonean sheath^10^, ^11^ at the right‐dorsal corner of the segment IV of the liver as illustrated in Figure 1, the D‐line theoretically lies on the edge of the extrahepatic major vasculobiliary sheath (shown in Figure S2). The tip of the dissecting forceps is visible through the posterior leaf of the gallbladder serosa outside of Rouviére's sulcus when the gallbladder is successfully isolated. Whenever the operator feels resistance at the tip of the dissection forceps, dissection is suspended to confirm the direction of the working forceps, and the procedure is resumed after confirmation of the dissection line to maintain the D‐line. After the serosa of the gallbladder at the opposite side of the D‐line was penetrated, surgical gauze was extracted through the dissected space. The gallbladder wall can usually be dissected away from the liver bed along the D‐line without difficulty when gallbladder wall scarring caused by cholecystitis is not severe. Practically, we make it a rule to first dissect the gallbladder along the D‐line within the subserosal layer and convert to subtotal cholecystectomy when the gallbladder wall is perforated despite gentle dissection; we regard this condition as severe scarring, which is inappropriate, and do not proceed to total cholecystectomy. Thus, the surgeon must consider carrying out a subtotal cholecystectomy (procedure is demonstrated in Video S1) instead of total cholecystectomy. Once the gallbladder is isolated along the D‐line by surgical gauze, CVS can be achieved without misidentification. By dissecting the cystic structure on the side facing the isolating gauze, it can be securely skeletonized into two cord‐like structures, namely, the cystic duct and the cystic artery (Figure 3). We named this procedure the segment IV approach for LC. The complete procedures for the D‐line method for difficult gallbladder and non‐difficult gallbladder are shown in Video S2, and Video S3, respectively.

Figure S3 shows the microscopic view of the resected gallbladder from Video S3, which indicates that the gallbladder is initially dissected on the D‐line within the subserosal layer of the gallbladder.

### 2.3 Distance between Rouviére's sulcus and D‐line

In the present study, we introduced the D‐line as a vectoral reference line along which the gallbladder is dissected safely. However, the relationship between the D‐line and Rouviére's sulcus must be clarified as to whether the D‐line can be used as a reference line for gallbladder dissection. To clarify this, in the present study, we reviewed 192 LC with segment IV approach including 62 difficult LC and 130 non‐difficult LC. Among them, 172 (44 patients with difficult gallbladder and 128 patients with non‐difficult gallbladder) achieved CVS and were included in the study. Distance between the D‐line and the roof of Rouviére's sulcus was measured in still pictures of the CVS (Figure S4). To ascertain the correct distance, the width of the 5‐mm forceps was referenced at such a position that the 5‐mm forceps applied on the D‐line and Rouviére's sulcus were in the same view. The distance between the D‐line and Rouviére's sulcus for each shape of the inferior surface of segment IV was obtained. Classification of the shape of the inferior surface of the quadrate lobe (segment IV) was used according to that reported by Rajkomar et al,^12^ which includes three shapes as rectangular, square or pyramidal by the length: width ratio of the inferior surface of segment IV.

### 2.4 Ethical considerations

This study was conducted in accordance with the Declaration of Helsinki with approval of the Ethics Committee of Jikei University School of Medicine (approval no. 30‐150 (9171)). All patients provided written informed consent prior to undergoing surgery.

## 3 RESULTS

**Figure 4:**
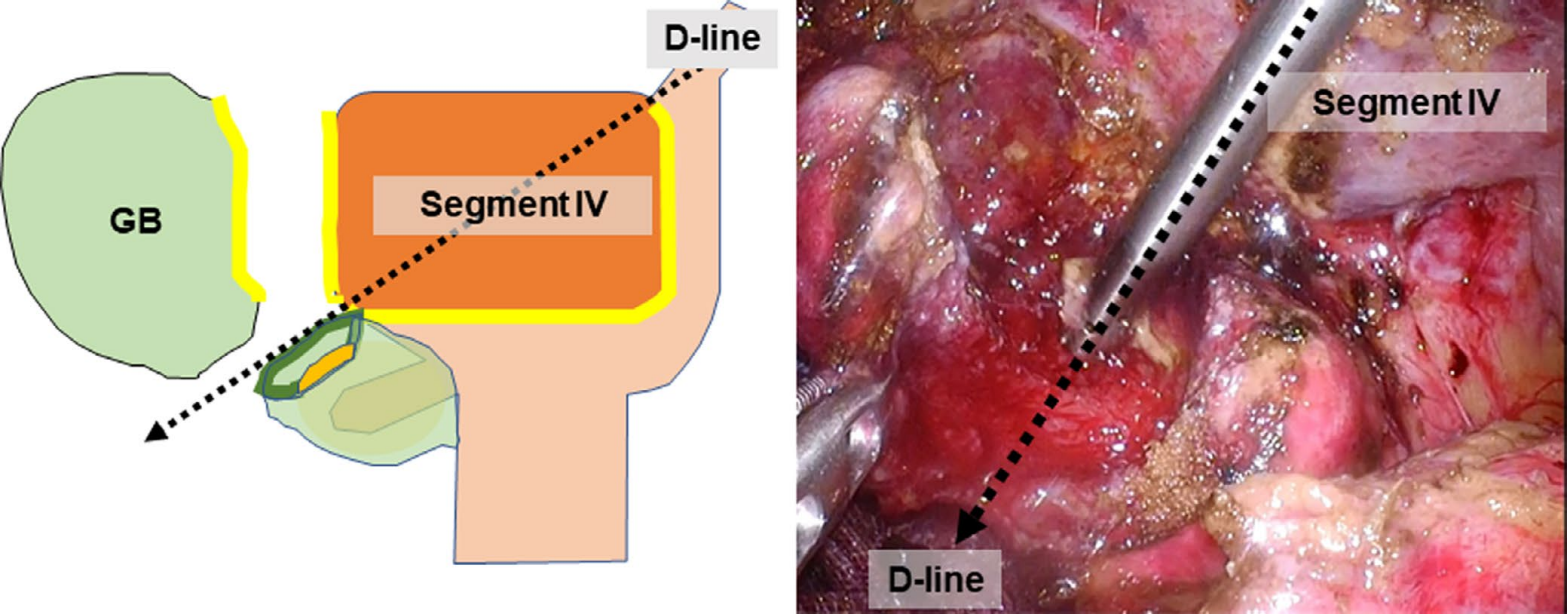
Conversion to subtotal cholecystectomy during the segment IV approach. When severe scarring makes gallbladder dissection along the D‐line difficult, bailout procedures (eg subtotal cholecystectomy) are carried out along the D‐line. D‐line, diagonal line of segment IV of the liver; GB, gallbladder

Patient characteristics, and physiological data of difficult and non‐difficult gallbladder are listed in Table S1. Intraoperative recognition rate of landmarks including Rouviére's sulcus, the base of segment IV and the D‐line and conversion rate to bailout procedure as well as postoperative outcome are summarized in Table S2. Successful gallbladder extraction along the D‐line was achieved in 44 (71%) patients with difficult gallbladder; total cholecystectomy and CVS were accomplished in all of these cases. The other 18 cases (29%) underwent subtotal cholecystectomy instead of LC because of the difficulty of gallbladder extraction along the D‐line (Figure 4). This difficulty was due to the presence of severe cholecystitis with inflammatory adhesion with surrounding structures. Intraoperative cholangiography was also carried out in these cases to investigate residual gallstones in the cystic duct before reconstruction of the remnants of the gallbladder. Median operative time and intraoperative blood loss were 135 (range, 54‐290) min and 10 (range, 0‐100) mL, respectively**.** No intra‐ or postoperative complications were seen in patients hospitalized. Mean postoperative hospital stay was 3.8 (range, 2‐5) days.

Shape of the inferior surface of segment IV was pyramidal in 46, rectangular in 94 and square in 32 cases. Mean distance between the D‐line and roof of Rouviére's sulcus were 4.6 (range 3.2‐5.6) mm in pyramidal, 7.3 (range 5.8‐9.4) mm in rectangular and 9.4 (range 7.6‐10.6) mm in square cases (Figure S5).

## 4 DISCUSSION

Although it was predicted that the VBI rate would decrease over time as the learning curve of LC flattened, the incidence of VBI remained steady at 0.5%.^13^, ^14^ Recent data suggest a declining trend in the occurrence of bile duct injury (0.32%‐0.52%) without any significant changes in the morbidity and mortality after LC.^15^ One explanation for the increasing risk of VBI may be misidentification; the common bile duct is commonly mistaken for the cystic duct; less commonly, an aberrant hepatic duct is misidentified as the cystic duct.^2^, ^3^ Thus, although the concept of CVS is useful for avoiding VBI due to misidentification, it is not always feasible for difficult LC for the following reasons. First, although severe‐grade cholecystitis is often accompanied by shrinkage of the hepatocystic triangle,^16^ the procedure used to achieve CVS also carries the risk of VBI. Second, separating the lower section of the gallbladder from the liver bed while achieving CVS is difficult, unless the cystic structure is divided.^17^ With such a background, TG‐18 recommends surgeons to consider a bailout procedure, such as subtotal cholecystectomy (rather than total cholecystectomy) without achieving CVS in difficult LC cases.^7^ In the present study, we proposed that the gallbladder is first extracted along the D‐line in order to secure an anatomical landmark for dissecting the cystic structures during difficult LC. This theory is based on an unchanged positional relationship between the root of the cystic plate and the right edge of the base of segment IV, regardless of the presence of cholecystitis. Therefore, the segment IV approach constantly isolates the gallbladder outside the hepatocystic triangle without encountering major vasculobiliary components. In the present study, the D‐line could be seen regardless of the shape of the inferior surface of segment IV and regardless of the grade of cholecystitis. The D‐line may run along the right border of the extrahepatic anterior sheath of the Glissonean pedicle and lateral to Rouviére's sulcus. Therefore, dissection along the D‐line was safely carried out and isolation of surgical gauze acts as the endpoint for dissection of the cystic structure, meaning that the surgeon will not misidentify the cystic structure and will be able to achieve CVS. In contrast, gallbladder perforation along the D‐line during dissection may be a sign of scarring of the gallbladder wall, which can result in VBI. In the case of scarring of the gallbladder wall, bailout procedures, such as subtotal cholecystectomy or open conversion, should be considered. In the present study, approximately 30% of the difficult LC cases were converted to subtotal cholecystectomy during gallbladder dissection along the D‐line in accordance with the decision criteria and at the discretion of the surgeon.

However, the segment IV approach does have some limitations. In cases where the margin of the gallbladder is hardly recognizable for anatomical identification of the D‐line because of inflammatory adhesion with surrounding structures, the segment IV approach is not applicable. In the present study, although we did not experience the condition where segment IV is unrecognizable, our operative policy is to convert to open surgery because the laparoscopic procedure of gallbladder dissection from the lateral side has a risk of injuring the anterior Glissonean sheath. Therefore, open conversion should be considered whenever the medial side of the gallbladder (segment IV) is obscure.

In conclusion, the segment IV approach is useful for deciding whether total cholecystectomy, open conversion, or other bailout procedures are necessary, depending on cholecystitis‐related gallbladder wall scarring in difficult LC cases.

## DISCLOSURE

Conflicts of Interest: Authors have no conflicts of interest or financial ties to disclose.

Author contributions: SF and HK participated in treating the patients, searching for literature, drafting the manuscript, and making the video. TM participated in treating the patients and analyzing the data. TH helped to analyze the data and participated in treating the patients. KY participated in planning the treatments. All authors read and approved the final manuscript.
