# Supplementary figures and images for: Segment IV approach for difficult laparoscopic cholecystectomy
Source: Ann Gastroenterol Surg. 2019 Nov 11;4(2):170–4. doi: 10.1002/ags3.12297 (PMC7105843; doi:10.1002/ags3.12297)

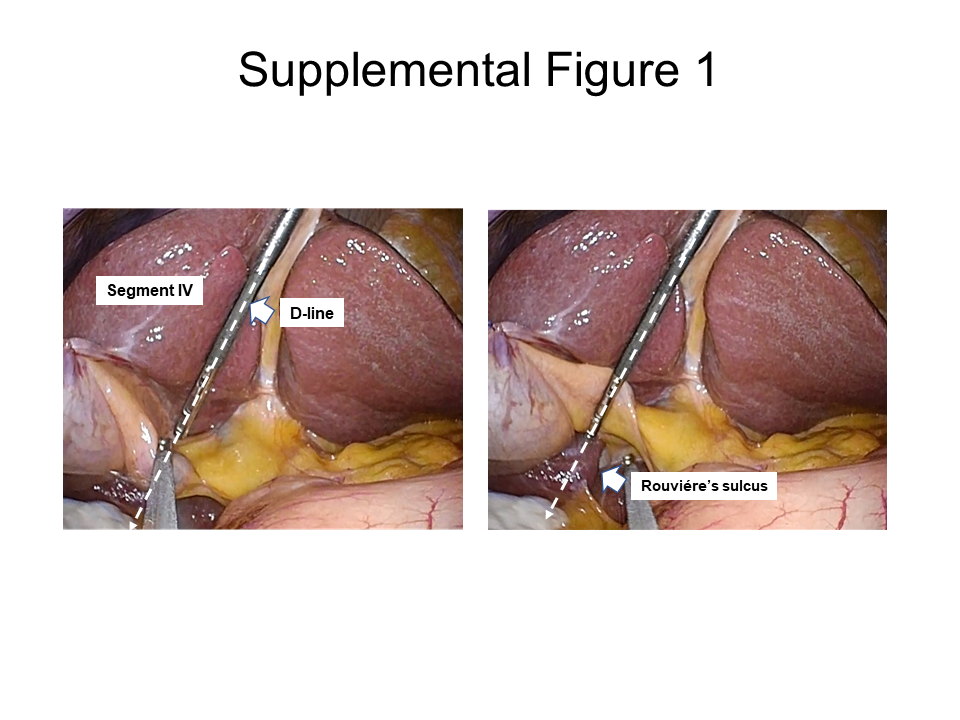

Supplement: Supplementary file 1 [file AGS3-4-170-s001.TIF]

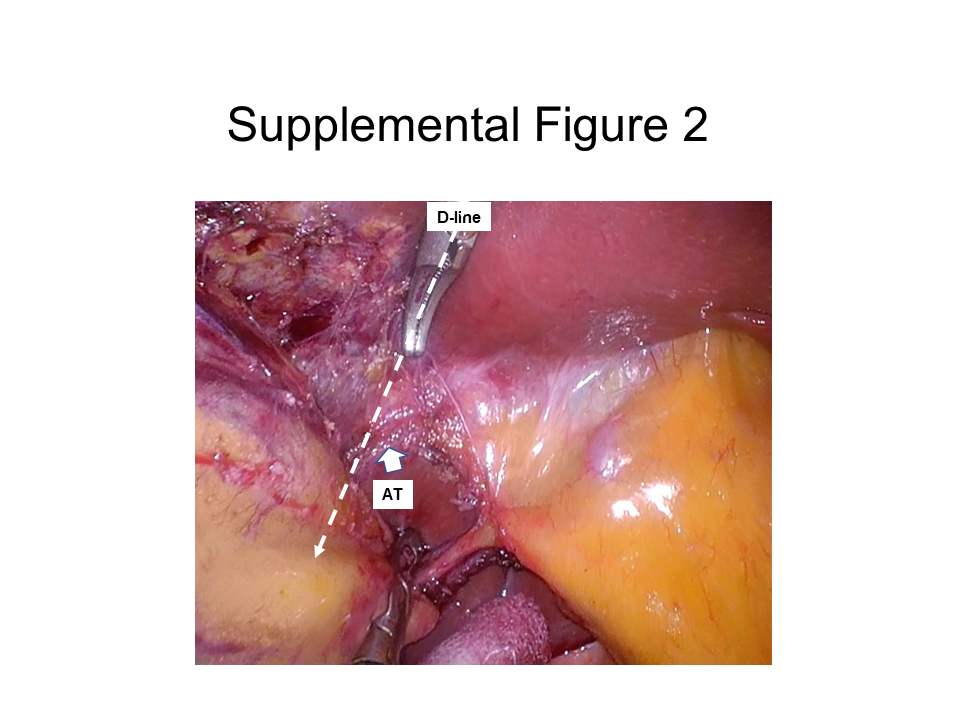

Supplement: Supplementary file 2 [file AGS3-4-170-s002.TIF]

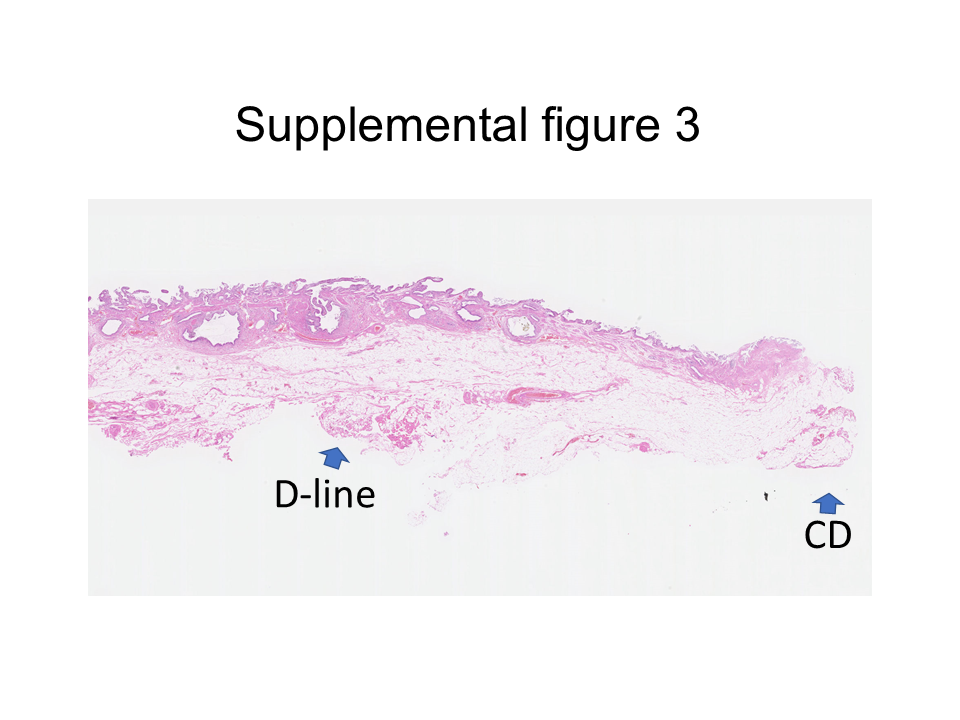

Supplement: Supplementary file 3 [file AGS3-4-170-s003.TIF]

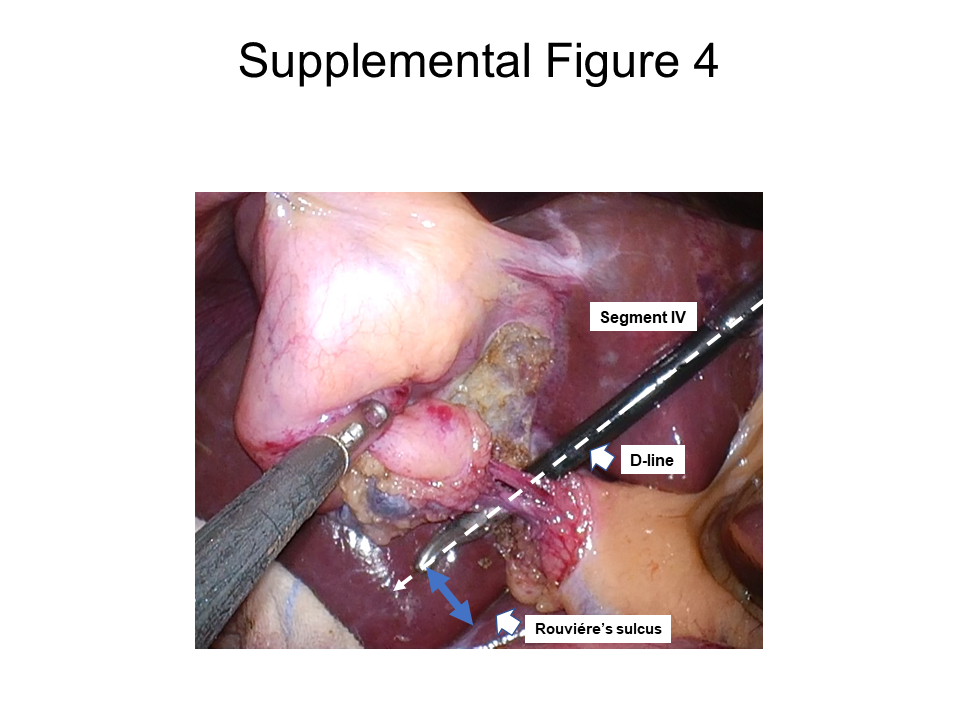

Supplement: Supplementary file 4 [file AGS3-4-170-s004.TIF]

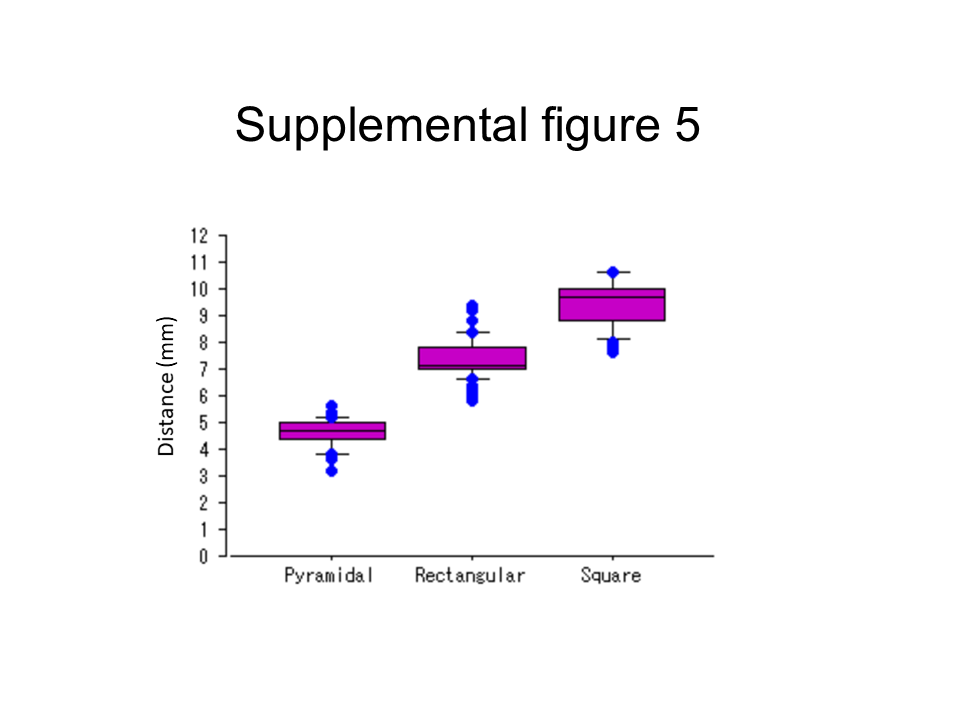

Supplement: Supplementary file 5 [file AGS3-4-170-s005.TIF]
