# Supplementary material for: Segment IV approach for difficult laparoscopic cholecystectomy
Source: Ann Gastroenterol Surg. 2019 Nov 11;4(2):170–4. doi: 10.1002/ags3.12297 (PMC7105843; doi:10.1002/ags3.12297)
Supplement: Supplementary file 6 [file AGS3-4-170-s006.docx]

**Supplemental Tables**

| Supplemental Table 1 Pathological and demographic data | | |
| --- | --- | --- |
|  | Difficult gallbladder (n=62) | Non-difficult gallbladder (n=130) |
| Male:Female | 22:40 | 58:72 |
| Mean age | 61 | 56 |
| Simple cholecystolithiasis | - | 68 |
| Gallbladder polyp | - | 26 |
| Acute cholecystitis (TG^†^ grade 0, 1) | - | 48 |
| Acute cholecystitis (TG^†^ grade 2, 3) | 62 | - |
| ^†^TG: Tokyo guidelines 2018 |  |  |
