# Supplementary material for: Segment IV approach for difficult laparoscopic cholecystectomy
Source: Ann Gastroenterol Surg. 2019 Nov 11;4(2):170–4. doi: 10.1002/ags3.12297 (PMC7105843; doi:10.1002/ags3.12297)
Supplement: Supplementary file 7 [file AGS3-4-170-s007.docx]

| Supplemental Table 2 Results of the segment IV approach | | |
| --- | --- | --- |
|  | Difficult gallbladder (n=62) | Non-difficult gallbladder (n=130) |
| Recognition of Rouviére’s sulcus | 55(89%) | 130(100%) |
| Recognition of base of segment IV | 62(100%) | 130(100%) |
| Recognition of the diagonal line of segment IV | 62(100%) | 130(100%) |
| CVS^†^ achievement | 44(71%) | 128(98%) |
| Conversion to subtotal LC^‡^ | 18(29%) | - |
| Conversion to open surgery | - | - |
| Conversion to conventional LC^‡^ | - | 4(3%)^§^ |
| Hemorrhagic complications | - | 1(1%) |
| Biliary duct injury | - | - |
| Incisional hernia | 1(2%) | 1(1%) |
| Superficial or deep incisional SSI^#^ | 2(3%) | 3 |
| Organ/space SSI | 1(2%) | 1(1%) |
| Mean operative time (min) | 135 | 75 |
| Mean blood loss (ml) | 10 | 3 |
| Mean postoperative stay (days) | 4 | 4 |
| ^†^Critical view of safety, ^‡^Laparoscopic cholecystectomy, ^#^surgical-site infections | | |
| ^§^Patients in whom visualizing the gallbladder was difficult due toobesity (BMI>30) | | |
